# A four-state Markov model of sleep-wakefulness dynamics along light/dark cycle in mice

**DOI:** 10.1371/journal.pone.0189931

**Published:** 2018-01-05

**Authors:** Leonel Perez-Atencio, Nicolas Garcia-Aracil, Eduardo Fernandez, Luis C. Barrio, Juan A. Barios

**Affiliations:** 1 Biomedical Neuroengineering research group (nBio), Systems Engineering and Automation Department of Miguel Hernandez University, Avda. de la Universidad s/n, 03202 Elche, Spain; 2 Unit of Experimental Neurology, “Ramón y Cajal” Hospital-IRYCIS, Carretera de Colmenar km 9, 28034 Madrid, Spain; 3 Biomedical Engineering program, National Experimental University “Francisco de Miranda”, Calle Norte, 4101 Falcon, Venezuela; Georgia State University, UNITED STATES

## Abstract

Behavioral states alternate between wakefulness (wk), rapid eye movement (rem) and non-rem (nrem) sleep at time scale of hours *i.e*., light and dark cycle rhythms and from several tens of minutes to seconds (*i.e*., brief awakenings during sleep). Using statistical analysis of bout duration, Markov chains of sleep-wk dynamics and quantitative EEG analysis, we evaluated the influence of light/dark (ld) changes on brain function along the sleep-wk cycle. Bout duration (bd) histograms and Kaplan-Meier (km) survival curves of wk showed a bimodal statistical distribution, suggesting that two types of wk do exist: brief-wk (wkb) and long-wk (wkl). Light changes modulated specifically wkl bouts, increasing its duration during active/dark period. In contrast, wkb, nrem and rem bd histograms and km curves did not change significantly along ld cycle. Hippocampal eeg of both types of wk were different: in comparison wkb showed a lower spectral power in fast gamma and fast theta bands and less emg tone. After fitting a four-states Markov chain to mice hypnograms, moreover in states transition probabilities matrix was found that: in dark/active period, state-maintenance probability of wkl increased, and probability of wkl to nrem transition decreased; the opposite was found in light period, favoring the hypothesis of the participation of brief wk into nrem-rem intrinsic sleep cycle, and the role of wkl in SWS homeostasis. In conclusion, we propose an extended Markov model of sleep using four stages (wkl, nrem, rem, wkb) as a fully adequate model accounting for both modulation of sleep-wake dynamics based on the differential regulation of long-wk (high gamma/theta) epochs during dark and light phases.

## 1 Introduction

The sleep-wake cycle (swc) is a dynamic phenomenon, resulting from complex interactions between the activity of neuronal populations in many brain structures, including the basal forebrain, hypothalamus and brainstem. Appropriate control of this brain activity permits behavioural state transitions between wk, rem and nrem sleep [1, 2]. Cycling between sleep and wk is regulated at time scales of seconds to several tens of minutes by the intrinsic activity of sleep-wake neuronal networks and of hours under control of circadian sleep rhythms [3, 4]. Main circadian oscillator regulating the brain arousal system is located in the suprachiasmatic nucleus [5, 6]. Molecules such as hypocretins (Hcrt), also called orexins, help to stabilize state changes. Loss of Hcrt in mice with a knockout of this gene leads to fragmentation of sleep, *i.e*., shorter wake and sleep bouts [7]. Thus, specific molecules may control not only the amount of sleep and wakefulness but also the maintenance of sleep and hence the bout length of different states.

Changes in the eeg are useful to distinguish wakefulness from deeper sleep stages, so that modifications of electrical activity are considered as biomarkers for predicting changes in the capability of animals to represent and respond to stimuli. Activity of Hcrt neurons is linked to heightened attentional states [8], and also to EEG frequencies in the gamma range (>30 Hz), a main characteristic of wk states [9–11]. Rodent waking is often divided into quiet and active wk based on whether the animal is still and quiet or exhibits exploratory movements. Transitions from quiet to active wake correlate with increased theta and gamma power, although detailed studies of activity changes in different modalities of wk and its relation with wake duration are lacking [12].

Existence of short and long bouts of sleep and wakefulness is a basic feature of sleep/wake control found in mammals [13]. Different duration of bouts of sleep and wake have been analyzed using survival curve analysis. This technique measures the probability that a given bout will survive long enough to reach a given duration, plotting the percentage of a state as a function of different bout length.

The resulting survival curves can be statistically analyzed to evaluate the sleep structure and underlying mechanisms, *e.g*. in rats [14], mice [15] or humans [16].

Although the presence of changes in sleep stages distribution during light and dark cycles is a clear indication of the existence of time-dependent variations in sleep architecture, transitions between sleep-wake states show a high degree of apparently random variability, suggesting that statistical tools should also be a useful way for studying sleep dynamics. Markov chains represent a class of stochastic processes of great interest for a wide spectrum of practical applications; in particular, discrete time Markov chains permit to model the transition probabilities between discrete states [17–20], allowing to model the dynamics of swc in mammals. Markov chains have also been used for studying physiology of human sleep [18], and some clinical applications of these methods have also been reported [2, 21–23]. For example, Markov chains have been used for studying polisomnographies of patients with narcolepsy, where a deficit of Hcrt produces a sleep with normal amounts of sleep and wake, but very brief states with increased transitions between them [2], findings also present in the knockout-Hcrt mouse model [24]. Given the stochastic nature of state transitions along the swc under normal and pathological conditions, swc are well suited to be modeled by discrete time Markov chains. Markov analysis provides accurate information of the probability of staying in one state *i.e*., state stability, closely related to state duration, and of the transition probabilities from and to that state, that cannot be obtained by other existing methods.

The current study was designed to describe the functional and EEG spectral differences between Brief and Long wk studying the modulation induced by dark/light changes over the sleep-wake pattern. Our data indicate that a better description of the sleep-wake architecture along ld cycle results extending the usual three-state model (wk, nrem, rem) to a four-state (wkl, nrem, rem, wkb) Markov model.

## 2 Material and methods

### 2.1 Animals

Male wild-type mouse (C57Bl/6) of three-month age were used in this study (N = 10). All experimental procedures were approved by Institutional Animal Care and Use Committee of Ramón y Cajal Hospital (Madrid, Spain) in accordance with Spanish (R.D. 1201/2005) and the Council Directive 2010/63EU of the European Parliament and the Council of 22 September 2010 on the protection of animals used for scientific purposes.

### 2.2 Surgery and EEG/EMG recordings

Animals were implanted under anesthesia with 1.5% isoflurane in 100% oxygen an electrode of nickel-chromium (140 microns) in prefrontal cortex (1.5 mm rostral, 1.5 mm lateral and 1 mm ventral to bregma), a second electrode in the CA1 region of hippocampus (-2.4 mm rostral, 1.5 mm lateral, 1.5 mm ventral), two stainless steel screws in the pre-frontal region, for ground and indifferent references, and a silver plate in the muscles of the neck for emg recording. Recovery took place in their cage in a sound attenuated chamber under 12:12h ld regime with a light intensity of 95 Lux and 0 Lux during the darkness phase, constant temperature 22–24°C and *ad libitum* access to food and water. Nine days after surgery, mice were transferred to a circular cage and the implanted cap fixed to a rotating anti-gravitational connector allowing free movements; ld regime was not changed (see S1 Fig). After a period of habituation of 72 h, 24 h of uninterrupted recording were acquired. eeg of the cortex, hippocampus and emg signals were filtered from 0.5 Hz to 200 Hz, amplified (x5000-10,000) (Cyberamp 380, Axon Instruments) and digitized at 1 kHz (Axon CNS Digidata 1440).

### 2.3 Sleep-wake staging

Sleep scoring was accomplished using an offline automated sleep scoring system, based on custom scripts (matlab 2008, Mathworks, usa). For automated staging, z-score of rms of band filtered eeg/emg signals was calculated from cortex; *δ*_*cx*_ (1-4 Hz), *σ*_*cx*_ (10-15 Hz), *β*_*cx*_ (15-25 Hz); hippocampus, *θ*_*hc*_ (4-10 Hz), $\gamma_{hc}^{1}$ (25-55 Hz); $\gamma_{hc}^{2}$ (55-125 Hz); and emg (55-90 Hz), and *θ*_*hc*_/*δ*_*cx*_ and $\beta_{cx}/\gamma_{hc}^{1}$ indexes were calculated. Initially, epochs with low $\beta_{cx}/\gamma_{hc}^{1}$, high *θ*_*hc*_/*δ*_*cx*_ and high emg are assigned to wk, epochs with low *θ*_*hc*_/*δ*_*cx*_, high $\beta_{cx}/\gamma_{hc}^{1}$ and low emg to nrem, and epochs with high *θ*_*hc*_/*δ*_*cx*_ and low emg to rem, using a fixed threshold, and non-assigned epochs were classified in a first pass of matlab K-*means* algorithm. Then, thresholds were recalculated and a second pass of the algorithm determined the definitive staging. This analysis classified every 5 seconds epochs in wk, nrem and rem states. Automated scoring was examined and visually corrected by two expert scorers. For helping in the analysis, quantified indexes of eeg/emg recordings were used by the scorers as an aid in ambiguous scoring epochs. After staging, the corresponding polysomnograms were constructed for each mouse recording, and mean duration and total amount of each state for 12 h of dark and light periods were calculated. For the visual analysis of the signals we used the software spike 2 (v 6.18, ced, uk).

### 2.4 Data analysis

After staging, analysis of recordings was performed using two complementary approaches: (1) for each state, bouts duration (measured to the nearest 5-sec epoch) were evaluated using survival curves, and (2) state-to-state transition probabilities were quantified using a Markov analysis.

#### 2.4.1 Survival curves

Bout durations of each recording were processed by Kaplan-Meier survival curve analysis using 5-sec (*i.e*. single epoch) time bins: *S*_(*ti*)_ = *S*_(*ti*−1)_*((*r*_*i*_−*d*_*i*_)/*r*_*i*_) where *S*_(*ti*)_ is the proportion of the original number of bouts surviving at the end of time bin *ti*, *S*_(*ti*−1)_ is the proportion of the original number of bouts remaining one time bin before *ti*, *r*_*i*_ is the number of bouts remaining at the start of time bin *ti*, and *d*_*i*_ is the number of bouts that terminate during time bin *ti*. Initially, the analysis was performed without reference to time-dependent factors (*i.e*., in each animal, all data were pooled; “24-h pooled”). To evaluate L/D modulation, light and dark epochs were then examined separately.

#### 2.4.2 Markov chains

A discrete time Markov chain is a sequence of random variables characterized by the Markov property, by which the state S at any time *t* + 1 depends on the state at time *t* but not on previous history. State transition probabilities describe the probability of going from stage i to stage j in a discrete time step (n) of 5 seconds: *P*_*ij*_ = *P*_*r*_(*X*_*n*_ = *j*|*X*_*o*_ = *i*). Wake epochs were separated in brief (shorter than 150 seconds) and long wakes (see Results); the transition probabilities of Markov chains between the 4 wk-sleep states (long wk, brief wk, nrem and rem) were arranged in a matrix with the form:

$$\left[\begin{array}{cccc} P_{\text{WKL}\to \text{WKL}} & P_{\text{WKL}\to \text{WKB}} & P_{\text{WKL}\to \text{NREM}} & P_{\text{WKL}\to \text{REM}}\\ P_{\text{WKB}\to \text{WKL}} & P_{\text{WKB}\to \text{WKB}} & P_{\text{WKB}\to \text{NREM}} & P_{\text{WKB}\to \text{REM}}\\ P_{\text{NREM}\to \text{WKL}} & P_{\text{NREM}\to \text{WKB}} & P_{\text{NREM}\to \text{NREM}} & P_{\text{NREM}\to \text{REM}}\\ P_{\text{REM}\to \text{WKL}} & P_{\text{REM}\to \text{WKB}} & P_{\text{REM}\to \text{NREM}} & P_{\text{REM}\to \text{REM}} \end{array}\right]$$

where each element of position (*i*, *j*) represents the transition probability *P*_*i*→*j*_: *e.g*., probability of transition from nrem state to rem state is denoted as *P*_nrem→rem_. State maintenance probabilities, (*p*_*ii*_) that describe the probability of remaining in one state, were denoted as *P*_wkl_, *P*_wkb_, *P*_nrem_, *P*_rem_. Markov chains have been already validated as a model for sleep dynamics in mouse and rat [14], so we did not perform specific tests to validate prior Markov analysis assumptions. Markov analysis was performed using the markovchain package (R environment, ver. 0.6.5.1) [20].

#### 2.4.3 EEG power spectral analysis

EEG signals were subjected to discrete Fourier transform to determine EEG power density spectra (0 to 120 Hz) for 5-s windows (Hamming function) with 0.20-Hz frequency resolution. Mean EEG spectral profiles for each behavioral state and time interval were calculated using artifact-free 5-s epochs. Time-course of activity in different bands was calculated by averaging power density in the corresponding frequency bins. To account for interindividual differences in absolute EEG power, power density in each frequency bin and for each state was expressed as a z-score of each individual mouse.

### 2.5 Statistical analysis

Data were presented as mean value or percentage of total value±se. Differences between the groups were evaluated using the Student’s t-test, Mann-Whitney test, and Bonfferoni test (Anova), depending on the compliance with the normality hypothesis of the data. In the case of the Kaplan-Meier survival curves the statistical analysis was performed with the log-rank test, using Sigmaplot (San Rafael, Hearne Scientific Software, 2006) and R statistical software [25].

## 3 Results

**Fig 1:**
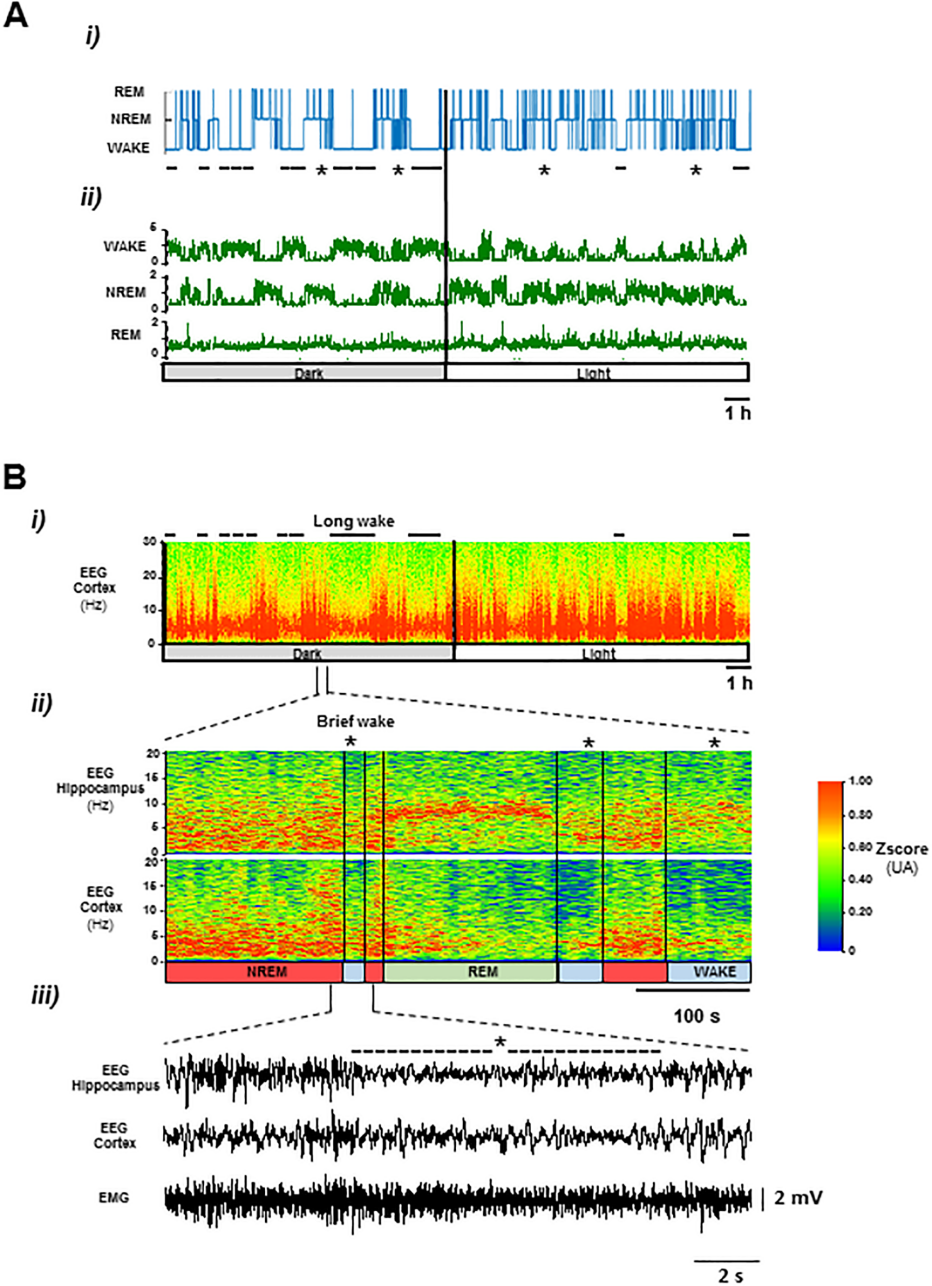
Representative data illustrating dark/light modulation of sleep-wake behavior in C57Bl/6 mouse. A i-ii) Hypnogram was generated by automatic sleep scoring of wk, nrem and rem sleep states. B) Long (-) and brief (*) wk bouts were identified based on the frequency pattern of cortex and hippocampal eeg activity (i-ii). iii) raw eeg during brief wk.

Transitions between behavioral states in adult mouse (wk, nrem, rem sleep) along the dark/light cycle are illustrated in Fig 1. Inspection of hypnograms shows clear differences between periods of dark and light along the swc: during dark (active) period, long lasting wks alternate with nrem-rem sleep cycles that contain brief and frequent awakenings; in light (resting) phase, brief awakenings dominate over long wks (Fig 1Ai and 1Aii). Such periods of long and brief wk (wkl and wkb) were also easily identifiable in the time frequency plots of cortex and hippocampus eeg activity (Fig 1Bi–1Biii).

**Fig 2:**
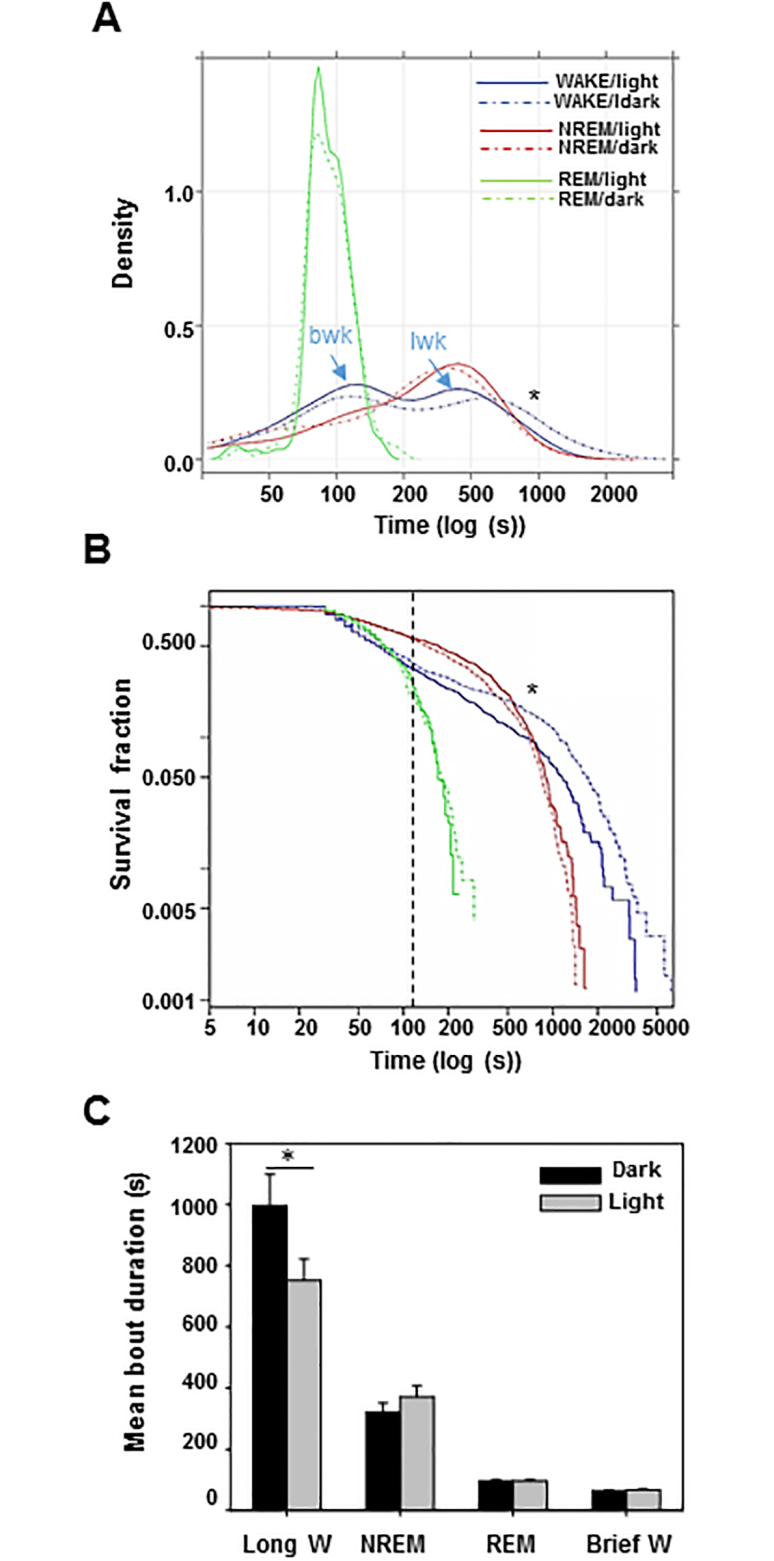
Statistics of wk, nrem and rem bout duration as a function of dark/light periods. A) Bouts duration histogram showed a clear bimodal density distribution of wake (brief- and long-wk) while nrem and rem distribution was unimodal; only the density of long-wk varied during circadian rhythm, lasting longer in the dark (active) than light (inactive) phase. B) Cumulative wk showed by Kaplan-Meyer survival curves exhibited a biexponential distribution (wkb and wkl, vertical bar) with a significant increment of wkl duration in dark phase. C) Mean bout durations of wkl nrem rem and wkb; duration increased in wkl during dark in comparison with light phase. Clear differences in duration distribution during light and dark period are shown (*, Anova and Log-Rank Test for Kaplan-Meier analysis *p* < 0.05).

Quantitative analysis of bout-duration bd for each state were obtained for dark and light phases. Histograms of wk bds showed, in both dark and light periods, the presence of a bimodal distribution corresponding to epochs of brief and long wk (wkb and wkl) (Fig 2A) separated by a cut-off value of 150 seconds, approximately. Interestingly, light and dark cycle modulation only affected to wkl, shifting its density peak toward longer lasting wk during dark phase (*p* < 0.05), in contrast to wkb bd distribution that did not vary significantly between dark and light periods. nrem- and rem-bd distributions approximated to unimodal distributions but neither changed significantly during the phases of dark and light. Inspection of the survival curve of wk drawn on double-logarithmic shows the presence of two linear segments with different slopes, suggesting a subyacent biexponential distribution while rem and nrem survival curves shown a unique segment, suggesting a monoexponential fit (Fig 2B). However, only the cumulative distribution corresponding to the longer wk duration of approximately 150 seconds enhanced during dark period. Thus, data clearly indicate that two types of wk can be segregated by its bout duration: long wakes (>150*s*) and brief wakes (<150*s*) and that only the long wk are under control of light and dark cycle (Fig 2D; *p* < 0.05).

**Fig 3:**
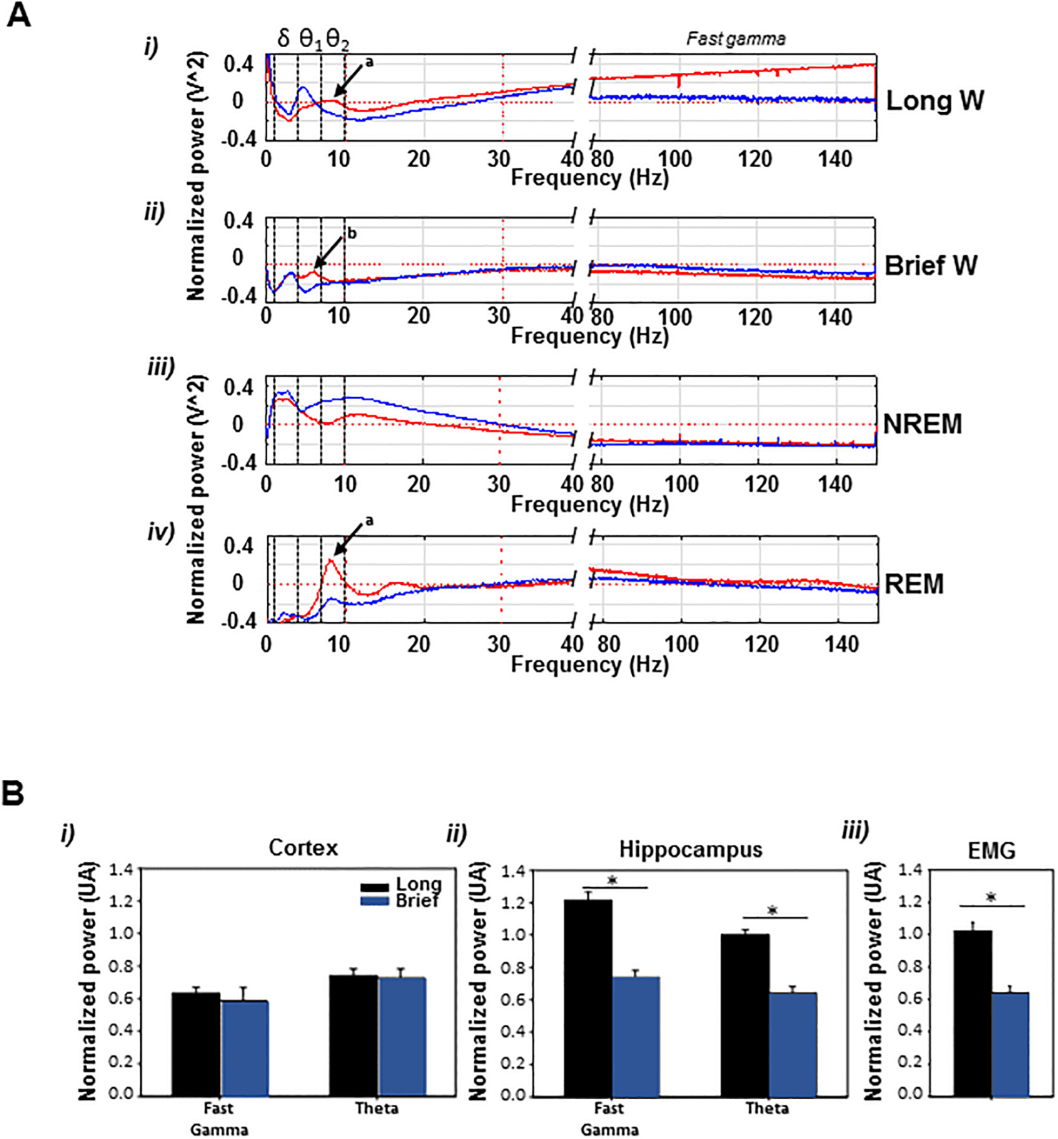
Spectral analysis of sleep stages normalized to the total spectrum of the signal in 24 hours. A) Hippocampal spectral content of long and brief wake is different (in red; cortex in blue); i and ii, long wake epochs had an augmented theta band with a characteristic 8 Hz peak (*θ*_2_ band; arrow a), whereas in the brief wake a lower frequency theta peak activity was at 6 Hz (*θ*_1_ band; arrow b); in addition, the power of fast gamma hippocampal frequencies in long wake epochs is significantly increased compared to brief wake. During nrem sleep a predominance of delta and beta power, and a reduction of gamma band were observed (iii) and in rem there was a predominance of theta (iv; arrow a) with low delta and beta, and an increased gamma band with respect to nrem. B) Long vs. brief wake spectrums; hippocampal, but not cortical, fast gamma and theta power (*θ*_2_) and EMG tone were decreased in brief-wk (*p* < 0.001).

Next, cortex and hippocampus EEG power spectra for brief and long wake stages were calculated using the *ad hoc*, empirically determined temporal cutoff of 150 s. Long-wk epochs had higher fast gamma power in hippocampus (CA1 region), higher hippocampal theta eeg power and increased emg tone than Brief-wk (Fig 3Ai-3Aii and 3B). Moreover, the theta frequency peak of 8 Hz that appears in hippocampus of waking mice (theta rhythm type I, usually associated to locomotion states) has a reduced power and slower peak frequency (6 Hz) in wkb epochs (theta rhythm type II, usually associated to alert immobility)(Fig 3A) arrows a and b in i and ii, respectively) [26]. No differences in fast gamma and theta in cortex were found. For completeness, we included in the figure the spectral density of nrem and rem epochs, where the expected increase of slow and beta power with gamma power reduction in nrem epochs and the increase of hippocampal theta power in rem epochs can be noticed.

The spectral differences found between both classes of wakes justify treating them as different states. Consequently, the dynamics of sleep-wake cycling were analyzed by applying a four-state (wkl, wkb, nrem and rem) Markov model.

**Fig 4:**
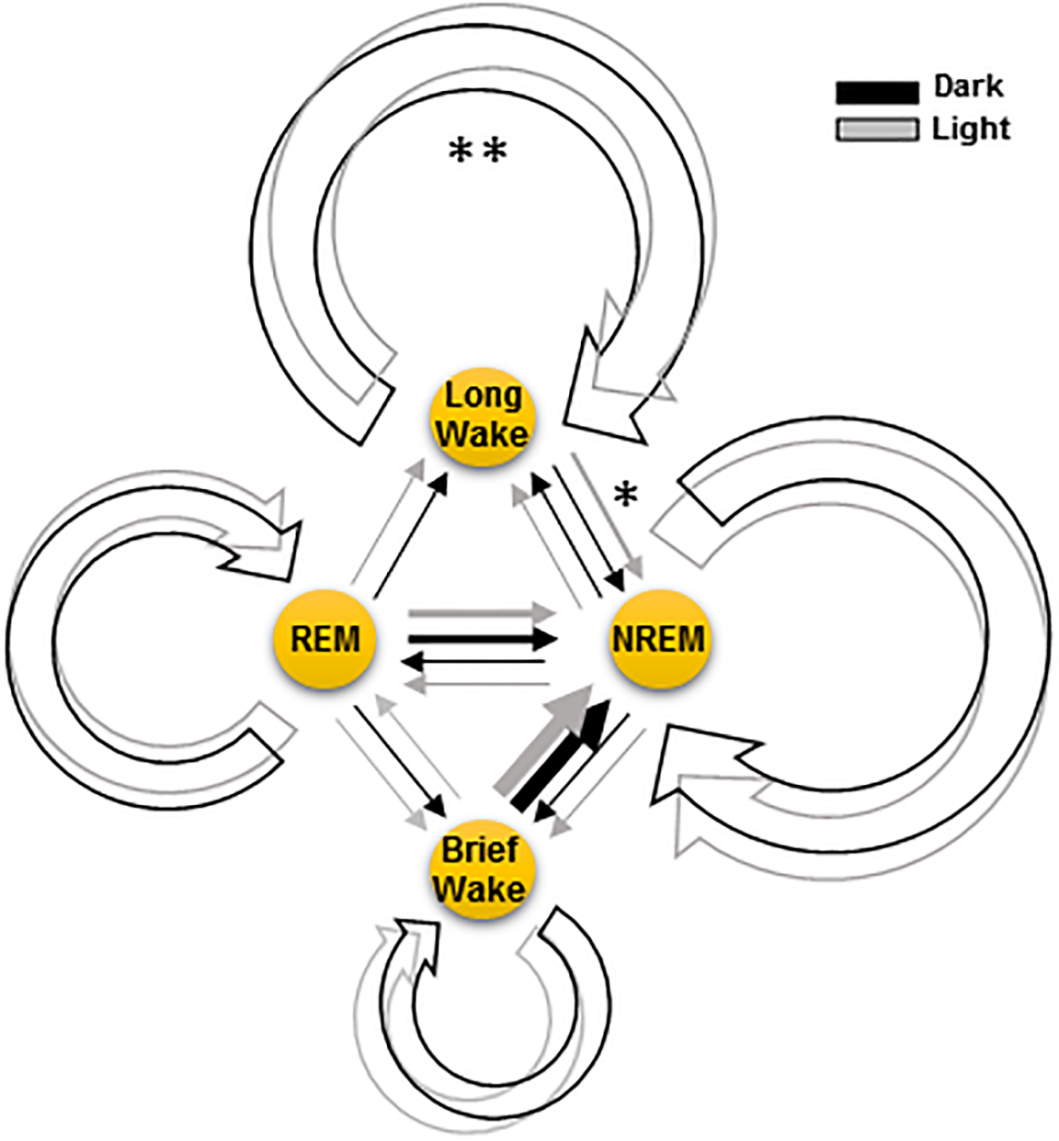
Diagram of four-state Markov model accounting for the wk-sleep dynamics across dark/light cycle. Circular arrows correspond to the probability of maintaining a state (i.e., the time spent in the corresponding state or bout duration), and straight arrows to transitions between states; arrows thickness are proportional to the corresponding probabilities (from Table 1), and the dark and light periods are represented in black and grey. The sleep-wake model of four states comprises of two wk states (with spectral differences, see text): a long-wk (wkl) and a brief-wk (wkb); and of nrem and rem sleep states. States of wkl and nrem were more stable than rem and wkb, while state transitions wkb to nrem and rem to nrem were the most probable. Circadian modulation increased the stability of wkl mainly by reducing the transitions from wkl to nrem during dark active period (*, *p* < 0.05; and **, *p* < 0.01).

Transition probabilities matrix derived from Markov analysis revealed high probabilities for staying in one state and much lower probabilities of transition between different states (see Table 1 and Fig 2). The order of state maintenance probabilities, which correlated with the corresponding values of bout-duration, was *P*_wkl_ > *P*_nrem_ > *P*_rem_ > *P*_wkb_, with statistically significant differences between all states (*p* < 0.001). State transitions between all behavioral states were theoretically possible, with the exception of ***p***_wkb→wkl_ by definition of states, the sequence of transition probabilities (*i.e*., the rate) was as follow: ***p***_wkb→nrem_ > ***p***_rem→nrem_ ≫ ***p***_wkl→nrem_ ≈ ***p***_nrem→wkl_ ≈ ***p***_rem→wkl_ ≈ ***p***_rem→wkb_ > ***p***_nrem→wkb_ > ***p***_wkl→rem_ > ***p***_wkb→rem_, revealing that there are important differences in the transitions probability depending on the involved states and its direction. Transitions from wkb and rem to nrem were the most frequent and statistically different each other and with the rest of state transitions (*p* < 0.001), while transitions of ***p***_nrem→wkl_, ***p***_rem→wkl_ and ***p***_rem→wkb_were more frequent than the transitions of ***p***_nrem→wkb_, ***p***_wkl→rem_ and ***p***_wkb→rem_ (*p* < 0.05). Regarding to dark/light modulation, only the stability of wkl state varied significantly (*p* < 0.01), increasing and decreasing respectively in dark and light phase, and only the probability of state transitions from wkl to nrem decreased significantly in the dark phase in comparison with light period (*p* < 0.05) (Fig 4).

**Table 1:** Probability matrix of transitions between states and of remaining in a state for discrete time steps of 5 seconds applying a four-state Markov model to fit the mouse sleep-wake dynamics along 12/12h dark/light cycle. Dark filled cells, dark period; light filled cells, light period.

	Long W	REM	NREM	Brief W
Long W	0.996289038	0.0001353482	0.003576614	0.00000000
REM	0.002403548	0.9638222661	0.030798463	0.002975273
NREM	0.003758884	0.0025628484	0.991916088	0.001762179
Brief W	0.000000000	0.000000000	0.059201561	0.940798439
Long W	0.994275350*↓	0.0001319087	0.005592741*↑	0.000000000
REM	0.003242978	0.9636607238	0.030080758	0.003015540
NREM	0.002976289	0.0027375917	0.992825566	0.001460553
Brief W	0.000000000	0.0012345679	0.058272307	0.949493125

*t*−student, *p* < 0.05 (*)

## 4 Discussion

The main findings of this report are that differences between long-wk and brief-wk in spectral profiles (Fig 3) and in their modulation by circadian ld changes (Fig 2) justify a new model of sleep-wake dynamics based in 4 states: long wk, brief wk, nrem and rem sleep.

Normal sleep is characterized by the cyclic alternance of nrem and rem sleep (*i.e*., the so called ultradian nrem-rem rhythm). In monophasic sleep, as in humans, after a first nrem-rem period, two to seven ultradian cycles follow until waking up. In polyphasic species, such as rodents, sleep is distributed across 24 h in hundreds of short bouts, including variable proportions of the two states, while apparently mostly beginning with nrem[27].

The regulation of swc in mammals is still a research topic [28]. Two-process model of sleep states that an homeostatic process (process S) interacts with a circadian pacemaker (process C) to regulate the swc [3, 29]. Main master clock of C is the suprachiasmatic nucleus, a small group of neurons located at the hypothalamus, influencing on Hcrt-containing wake-promoting neurons in lateral hypothalamus, that are the main responsible of regulating sleep and arousal [5, 6]. S process increases sleep pressure after long wk periods [6, 12]. Evidence now suggests that adenosine, a small inhibitory aminoacide, is the main candidate to be the physiological signal of S [30], although alternative theories do exist (see, for example, [31]). Brain levels of adenosine increase with prolonged wakefulness, initially in the basal forebrain and then throughout the cortex, and decrease during sleep[32]. Delta power of eeg in nrem sleep represents the principal marker of S during sleep, while theta activity in waking might be a marker of the rising limb of S [33]. Core body temperature and melatonin rhythms are markers of C [29]. The advances in neurophysiology have led to a proliferation of models of swc trying to extend it to a more physiological setting, *e.g*. the PR model and others [4, 34–37]; for review, see [38]). Although classical models of swc have been usually based on differential equations, discrete-time Markov chains have also demonstrated to be an adequate tool for modeling the cyclical dynamics of sleep and wk Markov analysis provides accurate information of the probability of staying in one state (*i.e*., the state stability, closely related to state duration), and of the transition probabilities from and to that state (*i.e*., rates) [2, 18, 20, 21, 24].

In addition to the regular sleep-wake pattern, humans and animals often exhibit brief awakenings from sleep [13]; brief awakenings are commonly observed across species and appear to occur randomly throughout the sleeping period, with a not fully understood dynamics. Because they exhibit robust scale-invariant features across different mammalian species, they may reflect intrinsic aspects of the endogenous sleep control mechanism [13]. In this work, we present a detailed analysis of wk bout duration in the mouse model. Brief-wk represented the majority of all wk bouts, but they were only slightly affected by circadian modulation, as has been previously reported in rats [14]. In contrast, the long wk bouts were strongly modulated by light and darkness (Fig 2).

The concept of short and long wk bouts differing in terms of their underlying mechanism and functional significance is not new [13–15, 37, 39] and differing statistical approaches have converged to this concept. Recent findings are clearly reinforcing the physiological role of Hcrt for sustaining long wk bouts [40, 41], while mechanism for brief wk bouts, usually treated in the context of arousal mechanisms are still under discussion (see, for example, [41], where an improved survival of brief wakes is described in orexin-tTA mice).

In our study, we have found, based on differences in modulation by light, that mice show a temporal breakpoint between both wk substates around 150 seconds. Previous reports on this topic, mainly reported in rats and using survival curves, conclude that both types of wk are present in rats [14, 39]. Simasko *et.al*. showed that sleep was more fragmented than previously recognized by using a cut-off interval of 5-min to separate brief and long wk. Light and dark cycle was not explored in [13], because they only used rodent data from light period. In our results, inspection of bd histograms and survival curves shown that 150 second is the temporal window wherein the effects of dark and light phases on wk diverge. This value, in mice, is comparable to theirs having in account species-dependent differences, and that some differences do exist in the methodology for sleep staging. In [40], it is reported the absence of Hcrt influences in short wk bouts (<1 min). Our findings, in WT mice, point to a longer threshold to differentiate between brief and long wk, maybe because light/dark modulation is the global result of the coordination of more systems than the hypocretinergic one, although more detailed experiments might refine this point.

We have also found spectral differences in eeg of hippocampus between wkl and wkb (Fig 3). Waking behaviours are usually subclassified into two substates; an active state associated with alertness and active exploration, where EEG is dominated by fast frequencies and theta rhythm in hippocampus, and a quiet state devoid of locomotion, where lower frequencies dominate (although always on higher levels than those of sleep states) [12, 42–44]. These spectral characteristics of active and quiet epochs are very similar to the findings in wkl and wkb that we report here, suggesting that this classification can be interpreted in the context of our framework. We have separately recorded eeg using deep electrodes in cortex and hippocampus, in order to clearly delineate function of both structures, avoiding the risk of volume-conducted propagation of electrical activity. Unexpectedly, we did not find significant differences in frontal cortical eeg activity in theta (7 − 10 Hz) and fast gamma (> 80) bands, between both kinds of wk. While hippocampus has a main role during open-field exploration, frontal cortical function is mainly related to temporal organization of behaviour and cognitive functions, activities that are severely reduced in a low-stimulation environment as we have used for our recordings. This might be a possible explanation of absence of differences in cortical function between wkl and wkb that might be explored in future experiments.

The analysis of the tp Markov matrix also provides some interesting insights about the properties of the swc. First, all behavioral states show a distinct intrinsic stability, quantified by their state maintenance probabilities, but they can be grouped into two highly stable states (wkl and nrem), and two very unstable states (wkb and rem). The most frequent transitions took place from the unstable states wkb and rem to the stable nrem *i.e*., while the probability of transitions between the most stable states (*i.e*., between wkl and nrem) or of rem to wkl and wkb are significantly less frequent; the rest of transitions, from nrem to wkb and from wkl and wkb to rem, were rare or nonexistent, respectively. Second, that only the stability of long wk state and its transitions to nrem are modulated by light: longer lasting wkl bouts are increased during active-dark period due to the reduction of transitions from wkl to nrem; during the resting-light phase, the probability of transitions from wkl to nrem is significantly higher than those in the opposite direction, *i.e*., from nrem to wkl. This finding clearly indicates that light/dark modulation affects to wkl-nrem cyclicity but not to nrem-rem-wkb sleep cycle (Fig 4).

The quantitative probabilistic relevance of transitions from the other states to nrem sleep seem to parallel the functional relevance of global slow oscillations generated in the whole brain along the NREM sleep [45]. The presence of ultradian or circadian regulation of rem sleep is still a matter of debate [46]. Bennington *et.al*. have proposed that the need for rem increases exclusively during nrem, thus suggesting a somehow subservient function of rem[47]. In [48], the role of intrasleep awakenings in the resetting of nrem/rem ultradian process is proposed for supporting this view. Recently, transitions from N2 to N3 sleep have been proposed as a regulator of rem sleep onset [49]. Other authors have postulated long-term and short-term homeostatic regulation of rem independent of nrem sleep with an accumulation in the absence of rem during both wk and nrem sleep [50]. Our data seem to support the first of the hypothesis, because we did not found modulation along light and dark cycle of probabilities for going to rem sleep (at least in the conditions of this experiment, where no deprivation or any experimental manipulation of sleep was performed), suggesting that every modification in rem amounts along the individual recordings are related to nrem changes. We also found that wkb-related probabilities were not modulated by circadian ld influences, so that wkb might be considered as an intrinsic part of the ultradian cycle nrem-rem, that maybe should be reclassified as nrem-rem-wkb cycle.

A key finding of this report is that transition probability from wkl to nrem is the main one modulated by light and dark cycle. Recently, the main role of gamma/theta-rich wk epochs in slow wave homeostasis has been reported [12], and we have demonstrated that these are also spectral characteristics of wkl. In our opinion, these findings clearly suggest a main role of wkl for increasing delta power in subsequent sleep, although experiments dissociating circadian modulation from rest-activity pattern would be necessary to further clarify the concept.

In summary, and based on reported findings, we propose a 4-state Markov model to fit experimental results. In such model superimposed on the swc is the nrem-rem-wkb sleep cycle with a preferential sequence:

$$\text{WKL}\to \overset{\leftarrow }{\text{NREM}⇌\text{REM}\to \text{WKB}}$$

## 5 Conclusions

In the mouse model, the application of quantified analysis of EEG and state bout duration in combination with transition probability analysis of sleep stages using discrete-time Markov chains constitute a powerful method for evaluating the probabilistic and statistical parameters of wk, nrem and rem sleep mechanisms, and for extension of sleep disorders. Using these tools, we found a bimodal distribution for duration of wk bouts (brief and long wk) with a temporal breakpoint of 150 seconds, that have a differential light/dark modulation and functional differences in eeg and emg (long-wk had higher emg tone, and hippocampal theta and fast gamma power). Between all observed state transitions, only those between long wk and nrem stages were modulated by light and dark cycle, favoring the hypothesis of the participation of brief wk into nrem-rem intrinsic sleep cycle, and the role of wkl in SWS homeostasis. In summary, we present an extended Markov model of sleep with four stages (long wk, nrem, rem, brief wk) able to fit sleep-wake dynamics and its modulation by light and darkness.

## Supporting information

S1 Fig A) Total amount of wk and nrem duration increased and decreased respectively during dark in comparison with light phase. B) Delta power modulation during light/dark periods indicatives of circadian entrainment.
(TIF)
