# Supplementary figures and images for: A four-state Markov model of sleep-wakefulness dynamics along light/dark cycle in mice
Source: PLoS One. 2018 Jan 5;13(1):e0189931. doi: 10.1371/journal.pone.0189931 (PMC5755762; doi:10.1371/journal.pone.0189931)

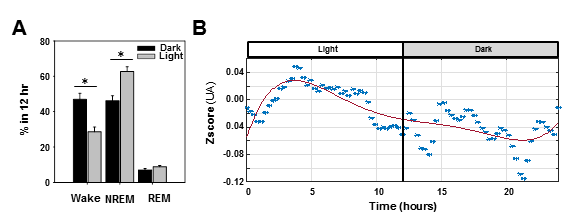

Supplement: S1 Fig — A) Total amount of wk and nrem duration increased and decreased respectively during dark in comparison with light phase. B) Delta power modulation during light/dark periods indicatives of circadian entrainment. (TIF) [file pone.0189931.s001.tif]
